# Multitissue ^2^H/^13^C flux analysis reveals reciprocal upregulation of renal gluconeogenesis in hepatic PEPCK-C–knockout mice

**DOI:** 10.1172/jci.insight.149278

**Published:** 2021-06-22

**Authors:** Mohsin Rahim, Clinton M. Hasenour, Tomasz K. Bednarski, Curtis C. Hughey, David H. Wasserman, Jamey D. Young

**Affiliations:** 1 Department of Chemical and Biomolecular Engineering and; 2 Department of Molecular Physiology and Biophysics, Vanderbilt University, Nashville, Tennessee, USA.

**Keywords:** Hepatology, Metabolism, Gluconeogenesis, Glucose metabolism, Intermediary metabolism

## Abstract

The liver is the major source of glucose production during fasting under normal physiological conditions. However, the kidney may also contribute to maintaining glucose homeostasis in certain circumstances. To test the ability of the kidney to compensate for impaired hepatic glucose production in vivo, we developed a stable isotope approach to simultaneously quantify gluconeogenic and oxidative metabolic fluxes in the liver and kidney. Hepatic gluconeogenesis from phosphoenolpyruvate was disrupted via liver-specific knockout of cytosolic phosphoenolpyruvate carboxykinase (PEPCK-C; KO). ^2^H/^13^C isotopes were infused in fasted KO and WT littermate mice, and fluxes were estimated from isotopic measurements of tissue and plasma metabolites using a multicompartment metabolic model. Hepatic gluconeogenesis and glucose production were reduced in KO mice, yet whole-body glucose production and arterial glucose were unaffected. Glucose homeostasis was maintained by a compensatory rise in renal glucose production and gluconeogenesis. Renal oxidative metabolic fluxes of KO mice increased to sustain the energetic and metabolic demands of elevated gluconeogenesis. These results show the reciprocity of the liver and kidney in maintaining glucose homeostasis by coordinated regulation of gluconeogenic flux through PEPCK-C. Combining stable isotopes with mathematical modeling provides a versatile platform to assess multitissue metabolism in various genetic, pathophysiological, physiological, and pharmacological settings.

## Graphical abstract

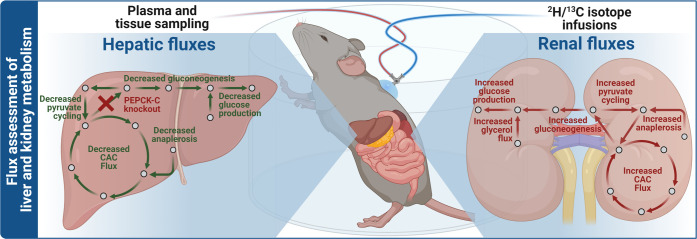

## Introduction

Biochemical methods to quantify gene transcript, enzyme, and metabolite levels are widely used to assess metabolic pathway regulation. Though informative and even vital in some contexts, static measurements of biomolecule abundance may not be reliable indicators of the movement of substrates through a metabolic pathway (i.e., metabolic flux). For example, cytosolic phosphoenolpyruvate carboxykinase (PEPCK-C) decarboxylates and phosphorylates oxaloacetate to form phosphoenolpyruvate (PEP); however, PEPCK-C expression does not solely determine the rate of hepatic gluconeogenesis (1). Burgess et al. have shown that PEPCK-C expression can vary widely while exerting limited control over gluconeogenic flux in perfused livers (1). Similarly, knockout of PEPCK-C from the liver (KO) increases the mRNA of several enzymes of the hepatic citric acid cycle (CAC; ref. 2); however, flux through the CAC is expected to be minimal under these conditions (1, 3).

Isotopic tracer techniques have been developed to address the limitations of static metabolite and enzyme measurements in order to more accurately quantify metabolic flux. In general, these methods introduce a stable isotope to a live biological system; metabolic fluxes are then determined by analyzing the isotopic enrichment of metabolites in that system using mathematical models (4, 5). Recent studies have applied metabolic flux analysis (MFA) to better understand nutrient production and utilization in normal and pathologic physiology (6–10). Several groups have focused on quantifying liver gluconeogenic and oxidative metabolism using stable isotopes, including our own prior contributions to assess in vivo fluxes in conscious, catheterized mice and rats (6, 11–15).

It may be reasonable and necessary to assume the kidney has a minor role in endogenous glucose production (V_EndoRa_) in some conditions, as no methods exist to disambiguate the hepatic and renal contributions to glucose production in conscious mice. As a result, the functional interaction between the liver and kidneys in controlling metabolic fluxes is largely understudied. For example, gluconeogenesis from PEP is severely impaired in perfused livers isolated from KO mice (1, 3), but rates of whole-body glucose production and gluconeogenesis are sufficient to maintain fasting euglycemia (2, 16). The authors note that 2 other potential sites of gluconeogenesis — the intestines and kidney — may compensate for the absence of hepatic PEPCK-C (16). Studies testing the significance of intestinal gluconeogenesis have been debated (17–19); a recent study by Potts et al. (20) showed that PEPCK-C in the small intestine supports nutrient processing of lipids and amino acids but is not necessary to maintain normal rates of glucose production during fasting. The kidney cortex expresses all gluconeogenic enzymes, including PEPCK, FBPase, and G6Pase and may significantly contribute to glucose production in certain contexts (21–23).

Here, we developed an isotopic flux modeling approach to simultaneously quantify gluconeogenic and oxidative metabolic fluxes in the liver and kidneys. This technique was then applied to WT and KO mice to test the hypothesis that renal gluconeogenic and oxidative metabolism compensates for deficiencies in hepatic gluconeogenesis in KO mice. Metabolic fluxes were estimated from the enrichment of plasma, liver, and kidney metabolites of WT and KO mice infused with ^2^H/^13^C isotopes. The results show that the kidneys have significant gluconeogenic potential upon loss of hepatic PEPCK-C. A 30-fold rise in renal gluconeogenesis was accompanied by an upregulation in the expression of both PEPCK-C and mitochondrial PEPCK (PEPCK-M) isozymes in the kidney. Furthermore, renal CAC fluxes were accelerated to sustain the energetic and metabolic demands of glucose production. More broadly, our multicompartment model provides a versatile platform to simultaneously assess in vivo hepatic and renal metabolism in a variety of experimental systems.

## Results

### Development of a liver-kidney multicompartment model to quantify glucose-producing fluxes in vivo.

**Figure 1:**
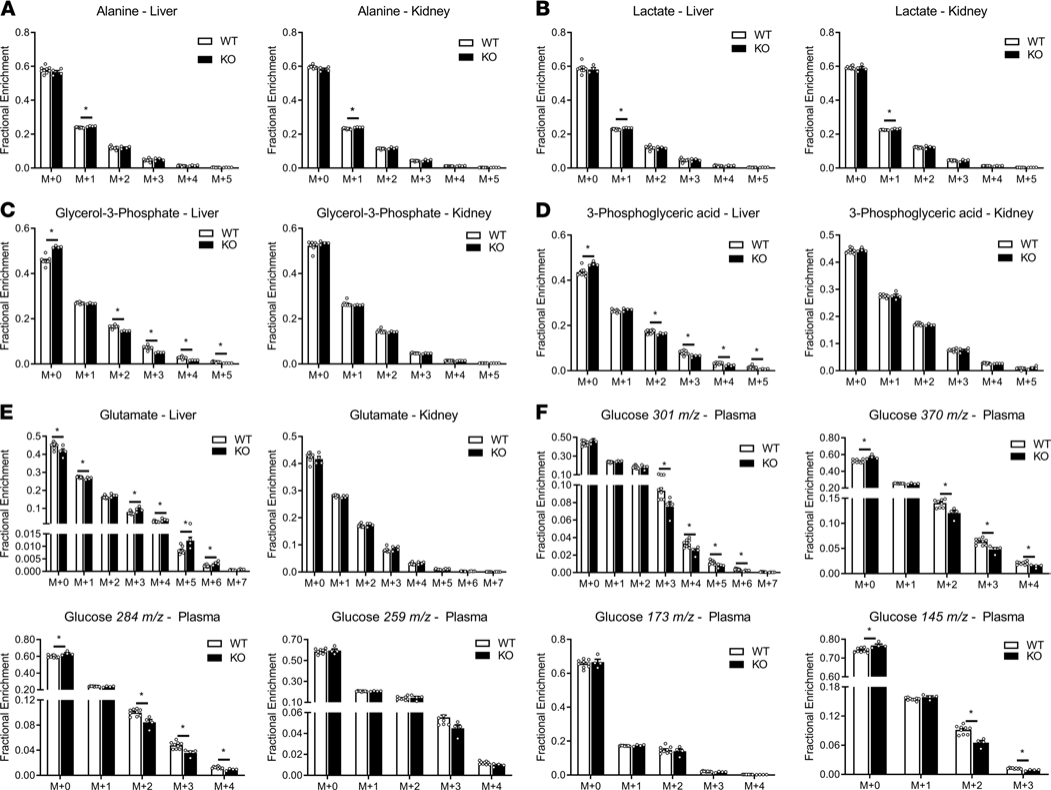
Mass isotopomer distributions (MIDs) of liver, kidney, and plasma metabolites. (**A**–**F**) alanine (**A**), lactate (**B**), glycerol-3-phosphate (**C**), 3-phosphoglyceric acid (**D**), glutamate (**E**), and plasma glucose (**F**) fragments from WT (*n =* 7) and KO (*n =* 4) mice. Differences between group means were assessed by a 2-tailed *t* test (*P < 0.05). Data are shown as mean ± SEM and are not corrected for natural isotope abundance.

To better understand the renal contribution to gluconeogenesis in the absence of hepatic PEPCK-C, a range of metabolites was isolated from the plasma, liver, and kidney of WT and KO mice obtained at the end of an infusion of ^2^H_2_O, [6,6-^2^H_2_]glucose, and [^13^C_3_]propionate. The mass isotopomer distribution (MID) of liver glutamate showed significantly higher enrichment in KO mice compared with WT mice, indicating that the livers of KO mice were able to extract the administered ^2^H/^13^C isotopes from plasma (Figure 1E). In contrast, metabolites measured above the PEPCK node, such as glycerol-3-phosphate (Figure 1C) and 3-phosphoglyceric acid (Figure 1D), exhibited an inverse trend with lower enrichments observed in the livers of KO mice. Arterial glucose enrichment in KO mice (Figure 1F) was also lower than in WT mice, suggesting higher contributions from unlabeled sources to plasma glucose production in KO mice. To assess hepatic and renal contributions in maintaining euglycemia in the absence of liver PEPCK-C, we developed a metabolic model (Supplemental Table 1; supplemental material available online with this article; https://doi.org/10.1172/jci.insight.149278DS1) to determine fluxes from MIDs of metabolites extracted from the plasma, liver, and kidneys (Figure 1).

**Figure 2:**
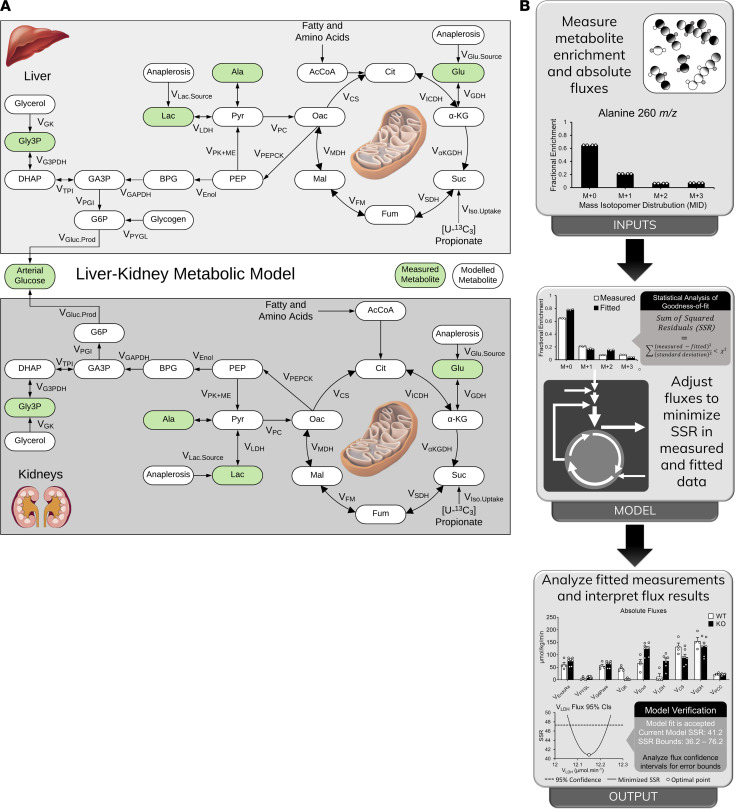
Liver-kidney multicompartment model enables quantification of tissue-specific fluxes using ^2^H/^13^C metabolic flux analysis (MFA). (**A**) Dual-organ metabolic network model developed for MFA. The top compartment shows the metabolic reactions in the liver, and the bottom represents those in the kidney. Measured metabolites are highlighted in green. (**B**) Overview of ^2^H/^13^C MFA workflow. Steady-state MFA typically has 2 experimental inputs: external uptake/excretion rates and metabolite enrichment measurements. These inputs are integrated into a metabolic model constructed using specialized software, such as INCA, which determines the best-fit flux solution by least-squares regression. Typical outputs from INCA include best-fit flux estimates for all metabolic reactions in the network, statistical analysis of the goodness of fit, and 95% CI for the estimated fluxes.

Our previously developed liver metabolic reaction network (11) was expanded to include a kidney compartment with reactions of glucose production and oxidative metabolism (Figure 2A and Supplemental Table 1, also see Methods). The resulting liver-kidney flux model was used to regress MIDs of measured metabolites (Figure 1 and Supplemental Table 2) and to obtain a best-fit solution for all fluxes in the metabolic network (ref. 24 and Supplemental Figures 1–3). The precision of hepatic and renal fluxes was determined by calculating a 95% CI for each estimated flux (Supplemental Figure 4). The multicompartment flux model yielded acceptable statistical fits with an average sum of squared residuals (SSR) of 56 ± 10 and 89 ± 8 for WT and KO groups, respectively. The goodness-of-fit was verified by comparing the SSR values with the expected range of the χ^2^ cumulative distribution function (95% CI, 55−108). The MFA approach is summarized in Figure 2B with further details available in Methods.

### Liver PEPCK-C KO mice exhibit significant renal gluconeogenesis compared with WT littermates.

**Figure 3:**
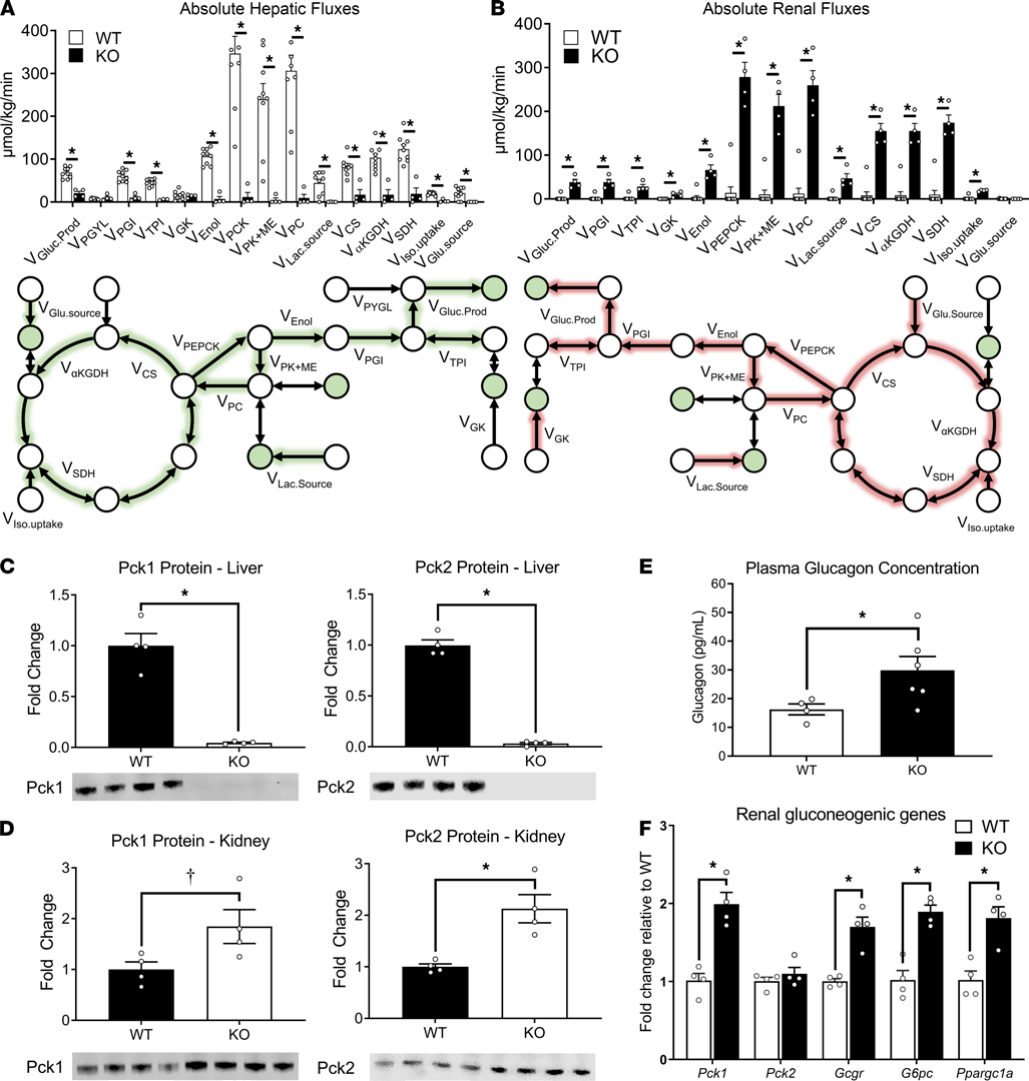
Liver PEPCK-C–KO mice exhibit significant renal gluconeogenesis compared with WT littermates. (**A**) Absolute hepatic fluxes for WT (*n =* 7) and KO (*n =* 4) mice, analyzed by a 2-tailed *t* test where *P < 0.05. The map represents the hepatic compartment shown in Figure 2B and Supplemental Table 1. Measured metabolic nodes are shown in green (Supplemental Tables 2 and 3). Arrows with green highlighting represent fluxes that are reduced in the livers from KO mice compared with WT littermates. (**B**) Absolute renal fluxes for WT (*n =* 7) and KO (*n =* 4) mice, analyzed by a 2-tailed *t* test where *P < 0.05. The map represents the renal compartment shown in Figure 2B and Supplemental Table 1. Measured metabolic nodes are shown in green (Supplemental Tables 2 and 3). Arrows with red highlighting represent fluxes that are increased in the kidneys from KO mice compared with WT littermates. (**C** and **D**) Pck1 and Pck2 fold change in the liver (**C**) and kidney (**D**) of KO (*n =* 4) relative to WT (*n =* 4) mice. Differences between group means were assessed by a 2-tailed *t* test (*P < 0.05, †*P* < 0.10). Protein expression was normalized to total protein content in each lane. (**E**) Plasma glucagon concentration after ~20 hours of fasting in WT (*n =* 4) and KO (*n =* 6) mice, analyzed by a 2-tailed *t* test where *P < 0.05. (**F**). Fold change in gene expression in kidneys of KO (*n =* 4) relative to WT (*n =* 4) mice, analyzed by a 2-tailed *t* test where *P < 0.05.

Fluxes estimated in KO mice using the liver-kidney model indicate diminished CAC activity and glucose production by the liver compared with WT mice (Figure 3A), consistent with previous results obtained in perfused livers (1, 3). Hepatic glucose production decreased from 70 to 25 μmol/kg/min in KO mice, and pyruvate cycling and enolase flux were diminished. The majority of glucose produced from KO livers came from glycogen (~40%) and gluconeogenesis from glycerol (~30%; Figure 3A), consistent with a significant decrease in enrichment measured in plasma glucose MIDs (Figure 1F). Furthermore, anaplerotic fluxes from glutamate (V_Glu.source_) and pyruvate (V_PC_) were significantly decreased, consistent with the reduction in total cataplerotic flux (V_PEPCK_) in livers of KO mice (Figure 3A). The increased enrichment of CAC intermediates and decreased enrichment of glycolytic metabolites in the liver — such as 3-phosphoglycerate — reflected the limited flux of labeled carbon exiting the hepatic CAC of KO mice (Figure 1, C–E).

The kidney compensated for the absence of hepatic PEPCK-C by increasing glucose production to maintain euglycemia (Figure 3B). Renal glucose production was increased from 2 to 34 μmol/kg/min through an acceleration in gluconeogenic fluxes from both glycerol and PEP. Oxidative, CAC flux also increased to sustain the energetic demands of gluconeogenesis. Cataplerotic and anaplerotic fluxes through PEPCK and pyruvate carboxylase (PC) were significantly higher in the kidneys of KO mice. Similar to WT hepatic metabolism, a large proportion (~75%) of renal cataplerosis was returned to the CAC through pyruvate kinase (and malic enzyme) flux (V_PK+ME_) in KO mice. To supply gluconeogenic precursors, increases in net anaplerotic fluxes from both propionate and lactate were observed in the kidneys of KO mice. A limited amount of net anaplerosis to the CAC from glutamine/glutamate was observed in kidneys, although significant exchange flux was detected between glutamate and α-ketoglutarate (α-KG; Supplemental Table 3).

Given that KO mice exhibited an increase in renal gluconeogenic flux from PEP, we hypothesized that expression of PEPCK also increased in the kidneys of KO mice. In the absence of hepatic PEPCK-C (Figure 3C and Supplemental Figure 5A), we observed an approximately 2-fold increase in PEPCK-C and PEPCK-M (Pck2) expression in the kidney (Figure 3D and Supplemental Figure 5B). These results are consistent with increased renal glucose production, gluconeogenesis, and PEPCK flux. Interestingly, immunoblots showed negligible protein expression of both liver PEPCK isoforms in KO mice (Figure 3C and Supplemental Figure 5A).

G6pc knockout in the liver increases renal gluconeogenic gene expression and circulating glucagon concentrations, leading to the hypothesis that glucagon-mediated changes in gene expression facilitate an increase in renal gluconeogenic flux (25). Based on these prior findings, we measured plasma glucagon concentrations (Figure 3E) and renal gluconeogenic genes regulated by glucagon receptor signaling (Figure 3F). Indeed, plasma glucagon levels were elevated in KO mice alongside increased kidney expression of the glucagon receptor (*Gcgr*), gluconeogenic enzymes (*Pck1, G6pc*), and mitochondrial metabolic transcription factor (*Ppargc1a*). Taken together, these data indicate that loss of cytosolic PEPCK in the liver leads to extrahepatic compensation from the kidneys in order to maintain euglycemia in vivo, potentially through increased glucagon action on the kidney.

### Results from the liver-kidney model are consistent with whole-body flux estimates and previous nuclear magnetic resonance–based (NMR-based) ^2^H/^13^C studies.

**Figure 4:**
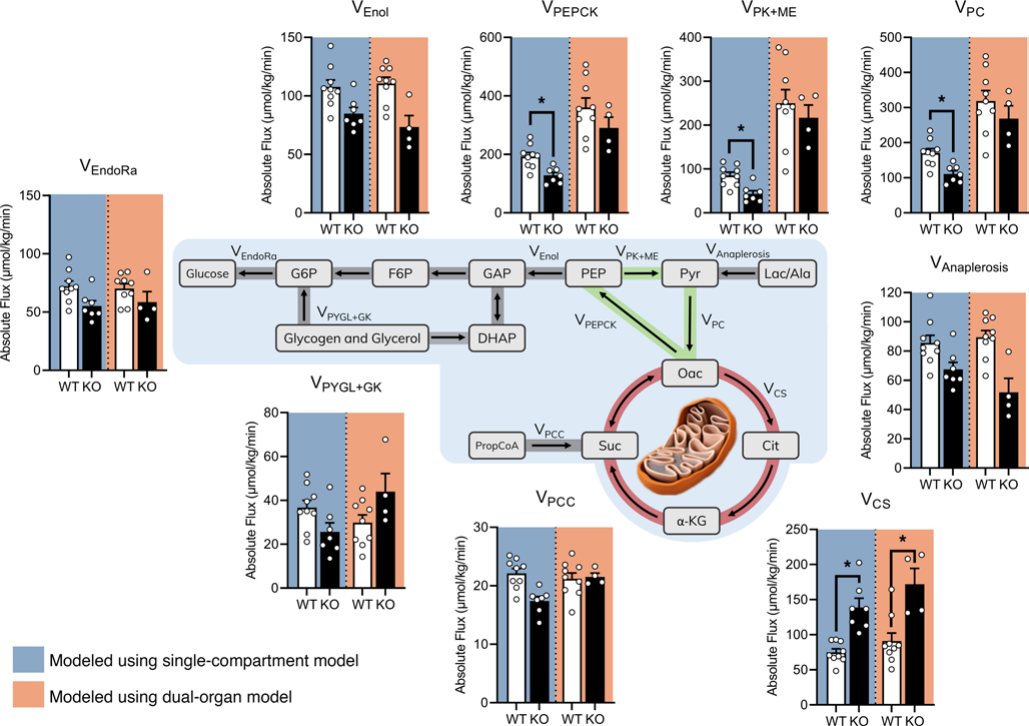
Comparison of flux estimates between the dual-organ model and a previously developed single-compartment model. Results from our previously developed single-compartment model (11), which estimates fluxes using plasma glucose enrichments only (blue), were compared with the sum of hepatic and renal fluxes estimated using the dual-organ model developed here (orange). Flux values are reported in μmol/kg/min (mean ± SEM) for WT and KO mice (*n* ≥ 4), analyzed by a 2-tailed *t* test where *P < 0.05. Pathways highlighted in green indicate a significant flux reduction in KO mice, whereas pathways highlighted in red indicate a significant flux increase in KO mice compared with WT littermates using the single-compartment model.

To further validate our results, we applied our previously developed MFA approach (11) to estimate whole-body gluconeogenic fluxes in WT and KO mice using only the measured plasma glucose MIDs (Figure 1F). The flux results obtained from our whole-body gluconeogenic model showed an approximately 86% increase in CAC activity and an approximately 33% decrease in pyruvate cycling in KO mice with no significant changes in V_EndoRa_ (Figure 4), consistent with results from prior NMR-based ^2^H/^13^C studies (16). Next, we compared these whole-body flux estimates with results from the multicompartment liver-kidney model. Since the whole-body flux model does not distinguish between hepatic and renal gluconeogenic contributions, we combined the liver and kidney flux estimates from our dual-organ model to obtain equivalent flux estimates. Summation of hepatic and renal fluxes showed strong agreement with whole-body flux estimates (Figure 4). Endogenous glucose production (V_EndoRa_), propionate uptake (V_PCC_), total anaplerosis (V_Anaplerosis_), net gluconeogenesis (V_Enol_), and contributions from glycerol and glycogen (V_PYGL+GK_) were comparable between the 2 metabolic models. Though not significant, the opposing trend in V_PYGL+GK_ observed between the 2 models may result from an improvement in the resolution of glycerol fluxes with the integration of glycerol-3-phosphate MID measurements. While the single-compartment model showed a decrease in V_PEPCK_, V_PK+ME_, and V_PC_, combined flux estimates from the dual-organ model showed no statistical differences in pyruvate cycling between WT and KO mice. This distinction likely stems from the use of tissue-specific alanine and lactate MID measurements in the dual-organ model, providing additional constraints to improve the resolution of pyruvate cycle fluxes. The combined magnitude of hepatic and renal fluxes were in general agreement with whole-body flux estimates (Figure 4) and with those measured by other groups (16).

## Discussion

**Figure 5:**
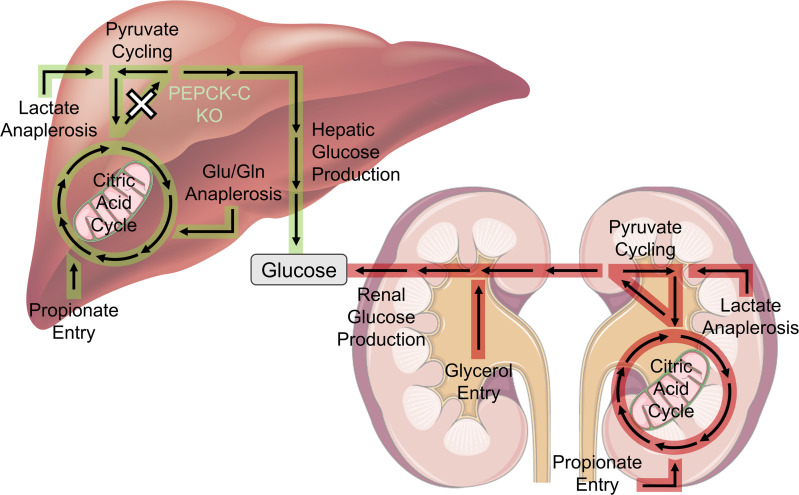
Metabolic pathways affected by knockout of hepatic PEPCK-C. Results from our liver-kidney metabolic model show that loss of hepatic PEPCK-C diminishes gluconeogenesis, CAC activity, anaplerosis, and pyruvate cycling in the liver. Increases in renal gluconeogenesis, CAC activity, and anaplerosis help maintain euglycemia during fasting. Pathways highlighted in green indicate a significant flux reduction in the livers of KO mice, whereas pathways highlighted in red indicate a significant flux increase in the kidneys of KO mice compared with WT littermates.

Prior studies have assessed the effects of liver PEPCK-C inhibition on hepatic gluconeogenic and intermediary metabolism in vivo or in perfused livers. However, no studies have introduced methods that separate the hepatic and renal contributions to gluconeogenic and oxidative metabolic fluxes in vivo. Here, we present a mathematical modeling approach that quantifies the metabolic contributions of the liver and kidneys to maintain glucose homeostasis. Similar to results shown in perfused livers (1, 3), KO mice had diminished hepatic gluconeogenesis, CAC activity, anaplerosis, and pyruvate cycling in vivo. The present study quantifies a previously undefined interaction whereby the kidneys of KO mice exhibited marked upregulation in gluconeogenic flux, anaplerosis/cataplerosis, and oxidative metabolism (Figure 5). These changes in renal fluxes correlated with an increase in the expression of downstream targets of glucagon signaling.

Fluxes calculated from the enrichment of plasma glucose alone have shown that whole-body glucose production is generally unaffected while gluconeogenesis is modestly reduced by the loss of hepatic PEPCK-C (16). Not only do we confirm those previous results, but we also characterize the metabolic flux compensation that occurs in the kidney to maintain euglycemia during inhibition of hepatic gluconeogenesis. Consistent with She et al., we observed a trend toward decreased V_EndoRa_ in KO mice, suggesting that the kidneys may not fully compensate for the loss of hepatic gluconeogenic capacity. The results here provide additional evidence that plasma glucose enrichment is reflective of liver metabolism under ordinary fasting conditions. Generally speaking, hepatic fluxes in WT mice estimated with the liver-kidney model are qualitatively similar to those determined from a single-compartment model of whole-body glucose metabolism (Figure 4). For example, total cataplerosis (V_PCK_) exceeds CAC-derived gluconeogenesis (V_Enol_), with surplus PEP returning to the CAC through pyruvate cycling (V_PK+ME_) in both models. CAC activity is also similar in magnitude to glucose production. Thus, approximations of hepatic metabolism from glucose enrichment alone may be reasonable in WT mice when more comprehensive techniques are unavailable or impractical.

When modeling both in vivo liver and kidney metabolism, removal of hepatic PEPCK-C diminishes liver gluconeogenesis, cataplerosis, anaplerosis, and CAC metabolism. These results are consistent with the fundamental coupling of energy-consuming, biosynthetic reactions and energy-producing reactions in the liver. Fluxes that deliver substrates for both oxidation and glucose synthesis are lowered when the liver is incapable of performing gluconeogenesis from the CAC. In fact, Berglund et al. have shown that fasting and glucagon administration lose their effects on the energy state of the liver when PEPCK-C is removed (26). PC and PEPCK are the prominent anaplerotic and cataplerotic nodes controlling the net flow of substrates in and out of the CAC and, thereby, regulate the initial steps of gluconeogenesis. PEPCK-C removal decreased hepatic pyruvate cycling, as V_PEPCK_, V_PK+ME_, and V_PC_ were diminished in vivo. It was recently shown that loss of PC from the liver significantly decreases the expression of PEPCK, reflecting a codependent coupling of gene expression and metabolite flux (13). In some conditions, cataplerotic activity of PEPCK promotes anaplerotic entry of carbon via glutaminolysis and uptake of glutamine (27). Our results show that the inverse relationship exists whereby loss of PEPCK-C decreases hepatic anaplerosis from all sources, including from sources that flux to α-KG (e.g., glutamate).

Until now, studies have relied on gene/enzyme expression or static metabolite assays to probe the impact of impaired hepatic gluconeogenesis on renal metabolic fluxes. Our study builds upon previous work (2, 25, 28) by quantifying the specific hepatic and renal flux contributions to glucose production in KO mice. Not only did the expression of gluconeogenic enzymes increase, but — more importantly — the rate of metabolic flux through renal gluconeogenesis and the CAC increased upon loss of liver PEPCK-C. Comparable with fluxes observed in WT livers, we observed significantly higher V_PEPCK_, V_PC_, and CAC fluxes in the kidneys of KO mice. These data are consistent with previous studies showing a reliance on renal glucose production during the anhepatic phase of liver transplantation in humans (29). A recent in vivo isotope labeling study reported increased enrichment of metabolites and expression of gluconeogenic genes in the kidney after knockout of hepatic PC, a major anaplerotic enzyme in the liver (13). It stands to reason that metabolite MIDs from that study (13) would be a rich data source to simultaneously regress renal and hepatic fluxes using the model developed here.

Cytosolic and PEPCK-M exhibit similar catalytic properties (30), yet PEPCK-M may only account for up to 5% of the total hepatic PEPCK activity in mice and rats (31, 32). It has also been reported that PEPCK-M requires the presence of PEPCK-C to substantially affect CAC activity and gluconeogenesis in perfused livers (33). The analyses presented here are consistent with these results, as the loss of PEPCK-C inhibited gluconeogenesis from PEP and significantly lowered PEPCK-M expression. This effect is similar to the relationship between Pdk1 and Pdk2 isozymes in the liver, where ablation of Pdk1 has a destabilizing effect on the protein levels of Pdk2 (34). In contrast, the expression of genes that mediate gluconeogenesis and oxidative metabolism in the kidney was increased as a result of hepatic PEPCK-C deletion. Some of these changes in gene expression may emanate, in part, through increased glucagon signaling in the kidney (25); the influence of other potential regulators (e.g., glucocorticoids and acidosis; refs. 25, 28) was not evaluated here. Interestingly, we observed increased renal expression of Pck2 protein but not *Pck2* mRNA in KO mice. Unlike PEPCK-C, it is less certain whether glucoregulatory hormones like glucagon and insulin influence PEPCK-M expression, which has generally been described as constitutive in nature (30, 35, 36). The results presented here suggest that renal PEPCK-M expression is posttranscriptionally upregulated upon the loss of hepatic PEPCK-C.

Recent work from others has helped characterize the contribution of glucose and other circulating metabolites to the CAC in multiple organs (7, 8). Though our work captures some systemic fluxes, it focuses more heavily on characterizing intermediary metabolism within the liver and the kidneys. We expect this approach to be leveraged to rigorously investigate liver-kidney interactions in models of diabetes, obesity, fatty liver disease, and steatohepatitis. One advantage of our methodology is that it does not depend on measurements of renal arterial-venous differences, which require additional surgical expertise and may introduce some analytical imprecision (17). Thus, this technology may provide new avenues to better understand the reciprocal or even pathophysiological relationship between the liver and kidneys in various contexts.

As detailed in Results, the dual-organ model relies upon assumptions that introduce some inherent limitations. The model does not account for additional sources of glucose synthesis other than the liver and kidney. While some studies indicate that glucose production by the small intestine is marginal in certain conditions (17, 20), our results do not strictly exclude the possibility of increased intestinal gluconeogenesis during a chronic deficiency in hepatic glucose production, as suggested elsewhere (28). If the intestines were to generate a significant amount of plasma glucose and exhibit a unique gluconeogenic metabolism, we would anticipate a significant lack-of-fit between simulated and measured isotopomers. However, statistically acceptable fits were obtained for all data sets in our study. That said, it is possible that the intestines have an undetectable (e.g., redundant) influence on whole-body adaptations to impaired hepatic gluconeogenesis that cannot be distinguished from kidney or liver contributions based on our available measurements. No other study has quantified in vivo renal glucose production and other associated metabolic fluxes in conscious, unrestrained mice. Thus, renal flux estimates reported here do not benefit from measurements obtained through arteriovenous balance and radio isotope–dilution methods and may not be reflective of cross-species differences (22, 37–40).

The dual-organ model estimated significantly different renal fluxes between WT and KO groups, yet a comparison of uncorrected MIDs showed similar enrichments for many kidney metabolites in WT and KO mice. This may be explained by the fact that MIDs are a composite of ^2^H and ^13^C enrichment, and as a result, a basic analysis of individual isotopomer patterns will not provide the resolution of a model-based flux regression. Furthermore, isotope incorporation into measured metabolite pools can result from a combination of net flux, as well as reversible/cyclic exchange flux of the tracer. Hence, many pools become enriched with isotope due to metabolite turnover in the absence of net flux through a pathway. The modeling software used in these studies (Isotopomer Network Compartmental Analysis [INCA]) provides a platform to rigorously test various modes of isotope incorporation while accounting for mass balance constraints on all pathway intermediates, thus enabling the detection of nonobvious changes in metabolic flux that could be overlooked by direct inspection of the mass isotopomer data (24). As a result, most fluxes were reasonably well resolved in both liver and kidney compartments.

The MFA approach used here relies on measuring a validated panel of labeled metabolites (Supplemental Table 2) that is sufficient to precisely quantify gluconeogenic and oxidative fluxes in our metabolic models. This targeted approach was not designed to assess global changes in adjacent or overlapping metabolic pathways, which might be revealed through an untargeted metabolomics analysis of tissue and plasma extracts. Lastly, a unique facet of our experimental system is the ability to measure plasma glucose enrichment over time and verify steady-state conditions over a similar isotope-infusion time course (11). However, end-point measurements of tissue metabolites can be obtained only in a terminal sample; therefore, steady-state assumptions for liver and kidney metabolites cannot be confirmed.

In summary, this study describes the development and application of a liver-kidney metabolic model that can be used to simultaneously assess intermediary metabolism in the liver and kidneys of individual mice, based on measurements of isotope enrichment in tissue and plasma metabolites. Our flux model shows that isotopic tracing and MFA are extensible tools that can aid in shaping our understanding of in vivo mammalian metabolism. Applying our dual-organ model, we show that mice lacking hepatic PEPCK-C maintain euglycemia by upregulating renal glucose production and oxidative metabolism. Loss of hepatic PEPCK-C diminishes gluconeogenesis, CAC activity, anaplerosis, and pyruvate cycling in the liver. Compensatory increases in expression of both PEPCK isozymes in the kidneys facilitates increased gluconeogenesis and cataplerosis from the CAC. Although hepatic PEPCK-C knockout has been extensively studied, its cross-regulatory effects on renal metabolism have not been rigorously defined. The integration of numerous measurements of metabolite enrichment from the plasma, liver, and kidney into a comprehensive liver-kidney metabolic model provides a platform to simultaneously evaluate hepatic and renal metabolism in vivo in other genetic, pathophysiological, physiological, or pharmacological contexts.

## Methods

### In vivo procedures in the mouse.

Mice with a liver-specific deletion of cytosolic PEPCK (*Pck1*) driven by the albumin-*cre* transgene (*Pck1*^lox/lox^Alb-*cre*) and WT littermates (*Pck1*^lox/lox^) were used (2). Male mice were studied to facilitate comparisons with previous studies on Pck1-KO mice. Mice were maintained on a 12-hour light-dark cycle with ad libitum access to water and a standard rodent chow diet (LabDiet 5001, PMI Nutrition International). Approximately 1 week prior to experimentation, jugular vein and carotid artery catheters were surgically implanted in 15-week-old PEPCK-C WT and KO mice for infusing and sampling, respectively (41). In vivo infusion studies were performed in long-term (~18 hour) fasted mice, similar to those described in detail elsewhere (11). Briefly, mice received a bolus of ^2^H_2_O to enrich body water at 4.5% and a primed (440 μmol/kg), continuous (4.4 μmol/kg/min) infusion of [6,6-^2^H_2_]glucose for 4 hours. A primed (1.1 mmol/kg), continuous (0.055 mmol/kg/min) infusion of [^13^C_3_]propionate (Cambridge Isotope Laboratories) was administered for ~2 hours prior to plasma sampling and tissue excision. Liver and kidney tissue were rapidly excised and freeze-clamped in liquid nitrogen at the close of the study. Plasma samples and tissues obtained at the end of the study were stored at −80°C prior to analysis.

### Gene expression analysis.

RNA was isolated from approximately 40 mg of powdered kidneys using TRizol reagent (Invitrogen, catalog 15596026) and RNeasy Mini Kit (Qiagen, catalog 74104), according to manufacturer protocols. cDNA was synthesized using the iScript cDNA synthesis kit (Bio-Rad, catalog 1708891) and diluted 10-fold with deionized water. cDNA was then combined with target primers (defined below; Integrated DNA Technologies) and iQ SYBR Green Supermix (Bio-Rad) and analyzed on a CFX96 Real-Time PCR System (Bio-Rad). Transcripts were quantified using the 2^–ΔΔCt^ method (42) and normalized to the WT group, with *Ppia* as an internal reference. Primer sequences were as follows: *Pck1*, forward 5′- CTGCATAACGGTCTGGACTTC -3′, reverse 5′- CAGCAACTGCCCGTACTCC -3′; *Pck2*, forward 5′- ATGGCTGCTATGTACCTCCC -3′, reverse 5′- GCGCCACAAAGTCTCGAAC -3′; *Gcgr*, forward 5′- TGCACTGCACCCGAAACTAC -3′, reverse 5′- CATCGCCAATCTTCTGGCTGT -3′; *G6pc*, forward 5′- CGACTCGCTATCTCCAAGTGA -3′, reverse 5′- GTTGAACCAGTCTCCGACCA -3′; and *Ppargc1a*, forward 5′- TATGGAGTGACATAGAGTGTGCT -3′, reverse 5′- CCACTTCAATCCACCCAGAAAG -3′. All abundances were normalized to *Ppia*: forward 5′- GGCCGATGACGAGCCC -3′, reverse 5′- TGTCTTTGGAACTTTGTCTGCAA -3′.

### Western blotting.

Protein was extracted from approximately 30 mg of frozen livers and kidneys with CelLytic MT mammalian tissue lysis/extraction reagent supplemented with protease inhibitor cocktail, and PMSF (catalog C3228, MilliporeSigma). Samples were centrifuged at 16,000*g* and 4°C for 20 minutes, and the resulting supernatants constituted the total protein extracts. Protein concentrations were determined by a BCA assay kit (Pierce BCA Protein Assay Kit, catalog 23225, Thermo Fisher Scientific). Samples were added in concentrations of 30 μg/lane for SDS-PAGE Western blotting using NuPAGE 10% Bis-Tris Mini Gels. Total protein on the membrane was quantified using Revert 700 Total Protein Stain (catalog 926-11016, LI-COR Biotechnology). Membranes were probed with antibodies against Pck1 (1:1000 dilution, catalog 10004943, Cayman Chemicals, RRID: AB_10141789) and Pck2 (1:1000 dilution, catalog 6924, Cell Signaling Technology, RRID: AB_10836185). All Western blots were imaged using the LI-COR Odyssey Fc imaging system, and signal was quantified using the LI-COR Image Studio software.

### Glucagon measurement.

Plasma glucagon was determined by the Vanderbilt University Mouse Metabolic Phenotyping Center (MMPC) Hormone Assay and Analytical Resources Core using an ELISA assay kit (catalog 10-1271-01, Mercodia Inc.).

### Metabolite extraction, derivatization, and gas chromatography–mass spectrometry (GC-MS).

Plasma glucose was extracted using cold acetone to precipitate protein. Samples were air dried at 60°C for 30 minutes, followed by immediate derivatization. Tissue metabolites were isolated from 30 to 50 mg of liver and powdered kidney using a biphasic methanol/water/chloroform extraction. The polar layer of the extract was isolated using a fine-tipped pipette and air dried overnight for storage at −80°C prior to derivatization. Plasma glucose samples were converted into 3 separate derivatives of di-*O*-isopropylidene, methyloxime pentapropionate, or aldonitrile pentapropionate according to protocols described elsewhere (43). Polar metabolites from tissue extracts were converted to their methoxime *tert*-butylsilyl derivatives (TBDMS) using MtBSTFA+1% TBDMCS (catalog 1-270144-200, Regis Technologies). Derivatized samples were injected onto a HP-5ms column (catalog 19091S-433, Agilent Technologies) in an Agilent 7890A gas chromatograph paired with an Agilent 5975C mass spectrometer. Data were acquired in scan mode, and metabolites were identified through comparison of mass spectra using a previously generated standard library. In some cases, multiple fragments of the same metabolite were used for flux analysis (Supplemental Table 2). Combining data from multiple fragments can improve the precision of flux estimates by providing increased information on the position of isotope labeling within a parent molecule. For example, the 3 glucose derivatives described above yield a total of 6 independent GC-MS fragment ions (301, 145, 173, 259, 284, and 370 *m/z*), which each contain a unique subset of carbon and hydrogen atoms derived from the parent glucose molecule (Supplemental Table 2). The accuracy of MID measurements was validated through comparison of the theoretical and experimental values of unenriched control samples.

### ^2^H/^13^C MFA.

The complete metabolic network and the carbon/hydrogen transitions used in the multicompartment liver-kidney model can be found in Supplemental Table 1. Metabolic equations were constructed from classical biochemical reactions and previously defined networks (6, 11). Glycerol-3-phosphate was included as a measurement to help resolve gluconeogenic flux from glycerol and to quantify the extent of equilibration between dihydroxyacetone phosphate (DHAP) and glyceraldehyde 3-phosphate (GAP) in the triose-phosphate isomerase reaction. Fumarate and malate measurements were also introduced, enabling the model to estimate the extent of equilibration within the 4-carbon branch of the CAC (6). Despite deletion of PEPCK-C in the livers of KO mice, the hepatic V_PEPCK_ reaction was included in the model to account for potential contributions from PEPCK-M and/or residual PEPCK-C expression in liver. The renal flux model was similar to that of the liver, except with respect to glycogen. Since the renal cortex does not synthesize appreciable amounts of glycogen (22, 23), glycogen synthase and phosphorylase reactions were omitted from the renal compartment.

MFA was performed by minimizing the SSR between model-simulated and experimental metabolite labeling measurements. The INCA software package (24) was used to develop metabolic models and regress all fluxes. Plasma glucose and polar liver and kidney metabolite MIDs were provided as measurements into INCA. The error in these measurements was set to either the root-mean square error of unenriched control samples or the standard error of measurement of technical GC-MS replicates — whichever was greater. Best-fit metabolic flux solutions were determined for each animal by least-squares regression of the experimental measurements to the isotopomer network model. To ensure that a global solution was obtained, flux estimations were repeated a minimum of 100 times from randomized initial guesses. A χ^2^ test was used to assess goodness of fit, and a sensitivity analysis was performed to determine a 95% CI associated with the calculated flux values. Initially, fluxes were estimated relative to the combined glucose production flux from liver and kidneys (V_Gluc.Prod_) constrained to an arbitrary value of 100. Relative fluxes were converted to absolute fluxes using the [6,6-^2^H_2_]glucose infusion rate and mouse weights.

### Statistics.

Data were analyzed using an unpaired 2-tailed Student’s *t* test without assuming a consistent SD between groups. Results with a *P* < 0.05 were considered significant.

### Study approval.

All protocols were approved by the Vanderbilt IACUC.

## Author contributions

CMH, JDY, and DHW designed research experiments. CMH, MR, and CCH performed the research experiments. TKB helped in running immunoblots. MR, CMH, and JDY analyzed data. MR, CMH, and JDY wrote the paper.

## Supplementary Material

Supplemental data

## Version 1 06/22/2021

Electronic publication
