# Supplementary material for: Multitissue 2H/13C flux analysis reveals reciprocal upregulation of renal gluconeogenesis in hepatic PEPCK-C–knockout mice
Source: JCI Insight. 2021 Jun 22;6(12):e149278. doi: 10.1172/jci.insight.149278 (PMC8262479; doi:10.1172/jci.insight.149278)
Supplement: Supplemental data [file jciinsight-6-149278-s175.pdf]

Supplementary Information to accompany:

Multi-tissue $^{2}H/^{13}C$ flux analysis reveals reciprocal upregulation of renal gluconeogenesis in hepatic PEPCK-C knockout mice

Authors and Affiliations

Mohsin Rahim[1,**], Clinton M. Hasenour[1,**], Tomasz K. Bednarski[1], Curtis C. Hughey[2,†], David H. Wasserman[2], Jamey D. Young[1,2,*]

[1]Department of Chemical and Biomolecular Engineering, Vanderbilt University, Nashville, TN 37212

[2]Department of Molecular Physiology and Biophysics, Vanderbilt University, Nashville, TN 37212

[†]Current affiliation: Division of Molecular Medicine, University of Minnesota, Minneapolis, MN, 55455

**These authors contributed equally

*Corresponding Author: Jamey D. Young

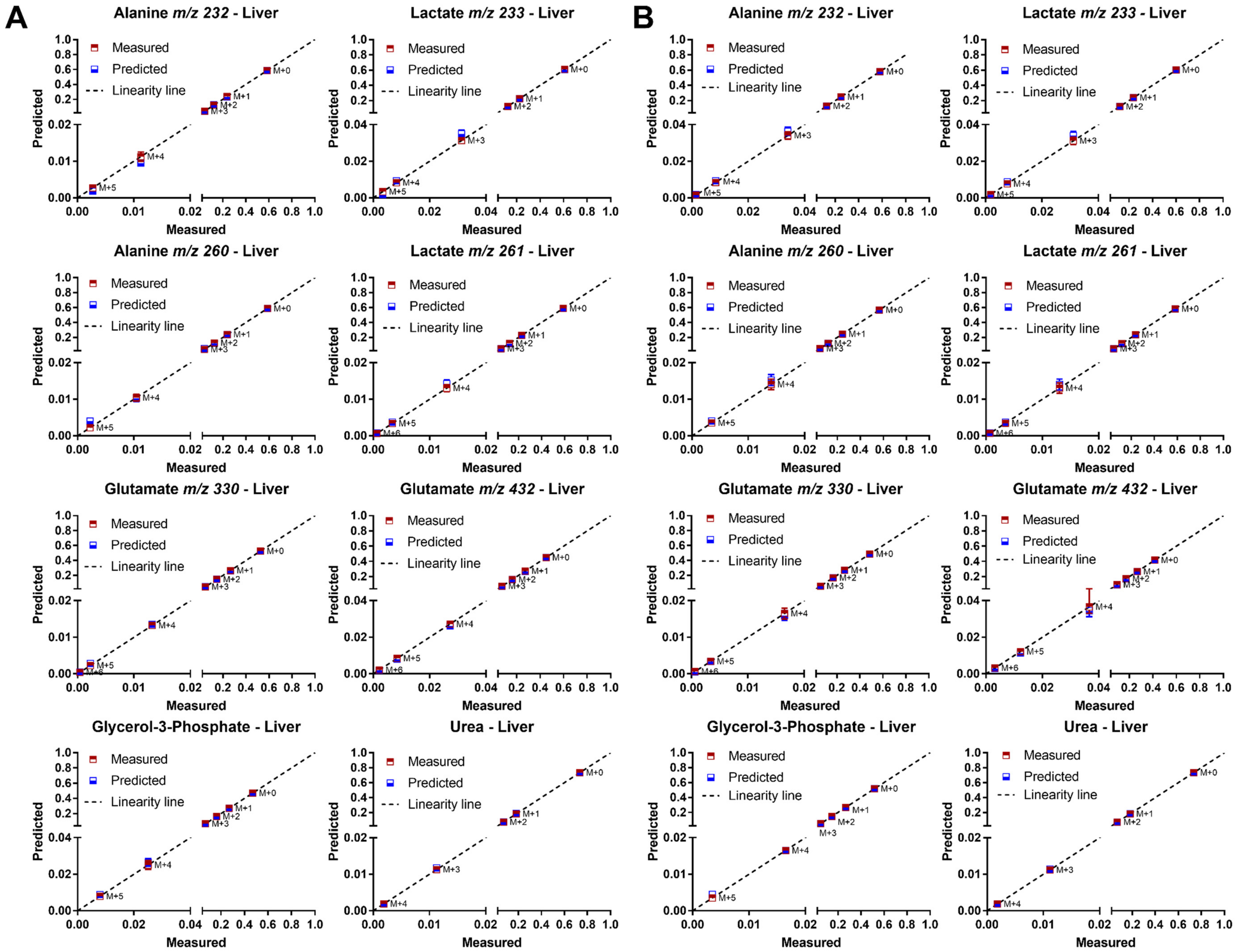

Figure S1. Measured and predicted fractional enrichments of liver metabolites for WT and KO mice. Linearity analysis of measured and predicted liver metabolite MIDs for **A.** WT (n=7) and **B.** KO (n=4) mice. Values on the x and y axes (means±SEM) represent the uncorrected measured and predicted fractional enrichments, respectively.

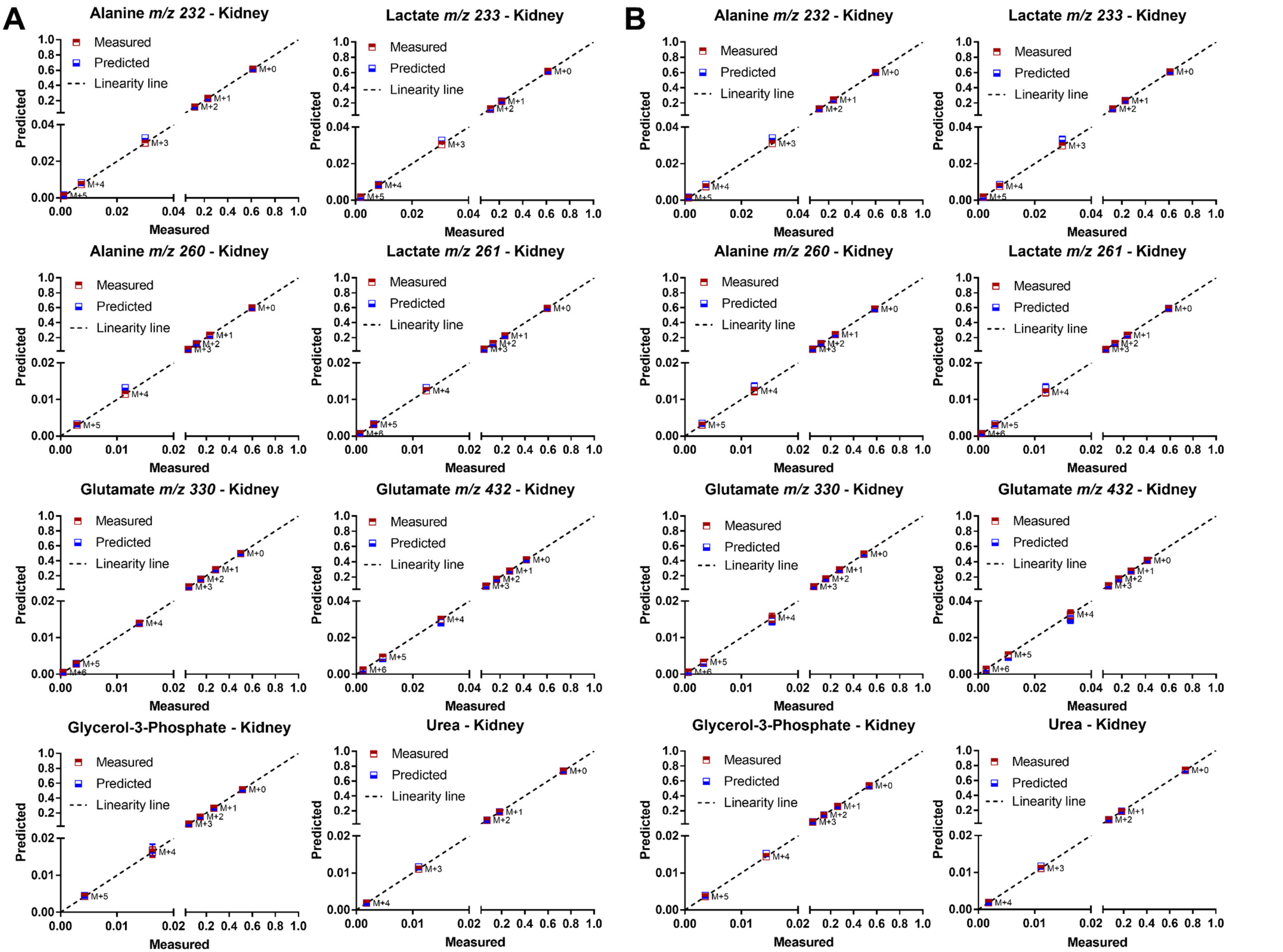

Figure S2. Measured and predicted fractional enrichments of kidney metabolites for WT and KO mice. Linearity analysis of measured and predicted kidney metabolite MIDs for **A.** WT (n=7) and **B.** KO (n=4) mice. Values on the x and y axes (means±SEM) represent the uncorrected measured and predicted fractional enrichments, respectively.

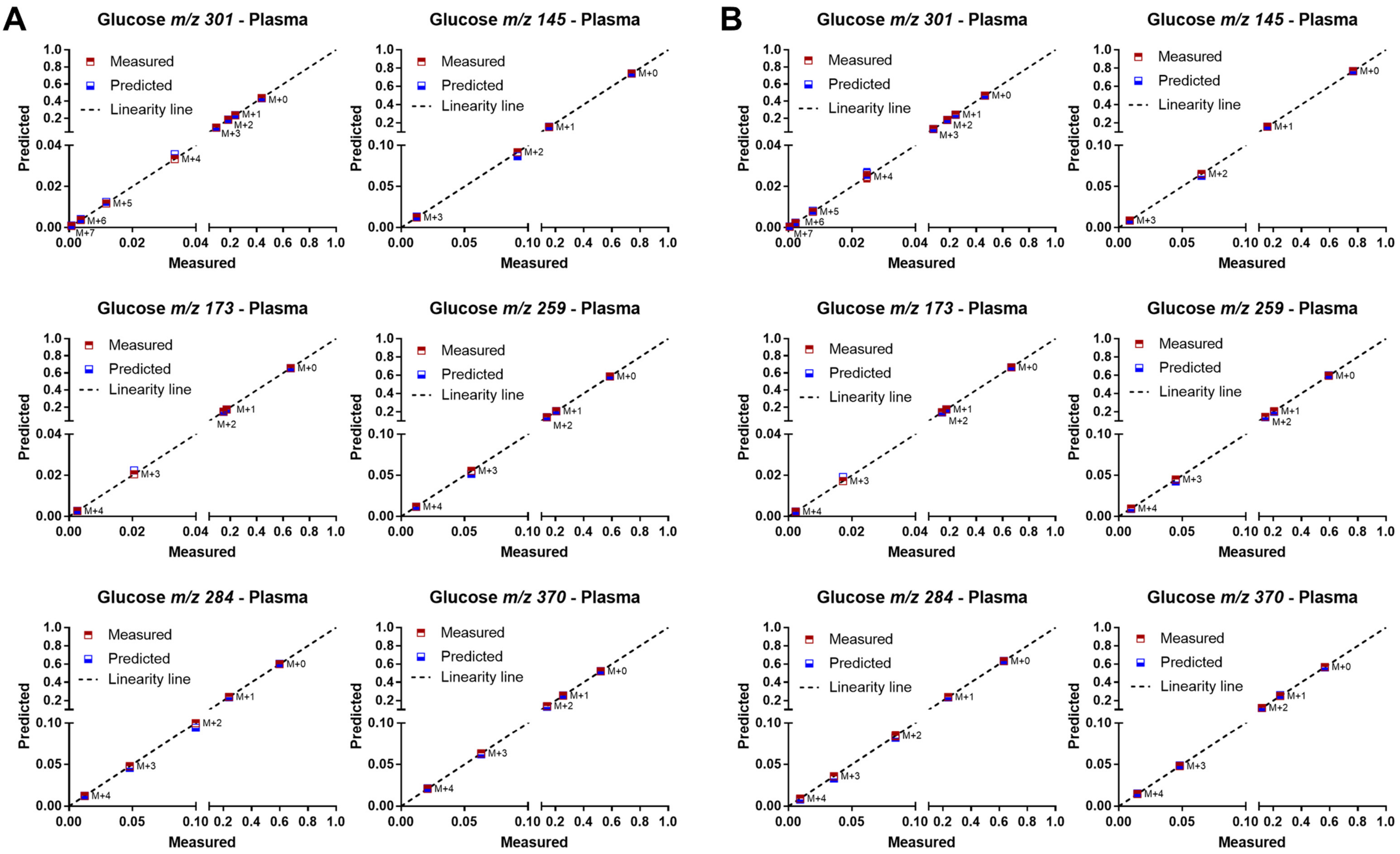

Figure S3. Measured and predicted fractional enrichments of plasma glucose for WT and KO mice. Linearity analysis of measured and predicted plasma glucose MIDs for **A.** WT (n=7) and **B.** KO (n=4) mice. Values on the x and y axes (means±SEM) represent the uncorrected measured and predicted fractional enrichments, respectively.

A

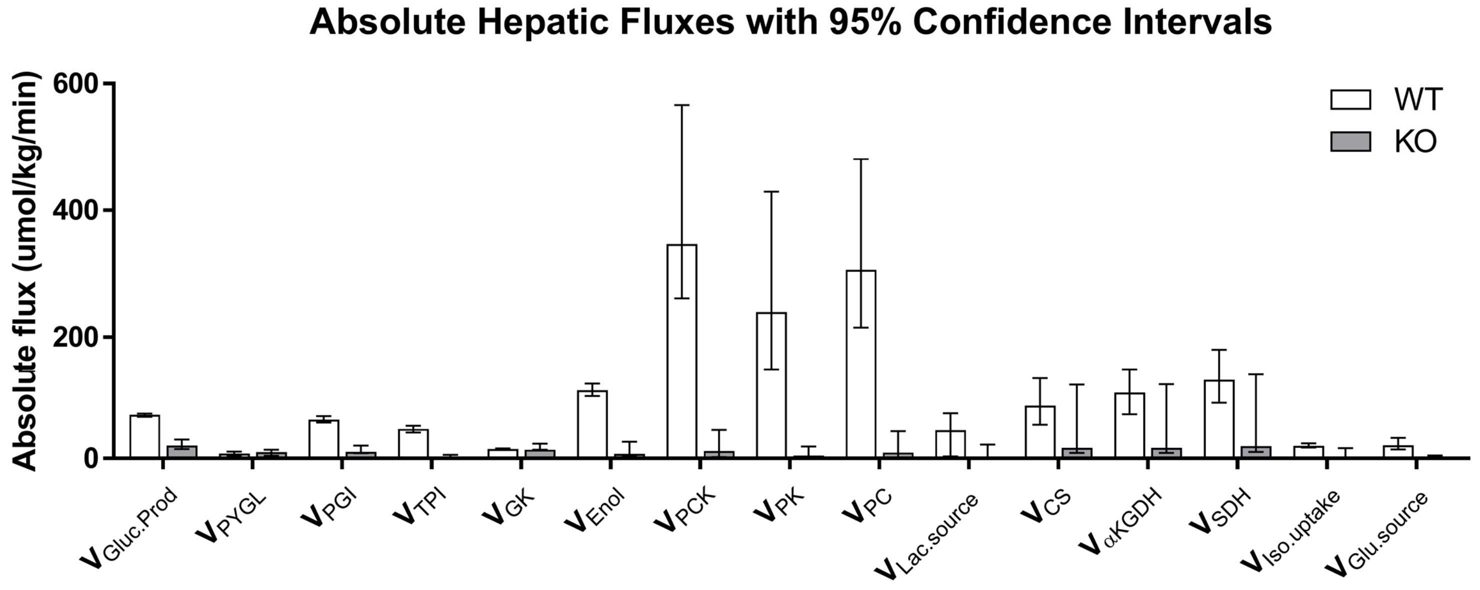

B

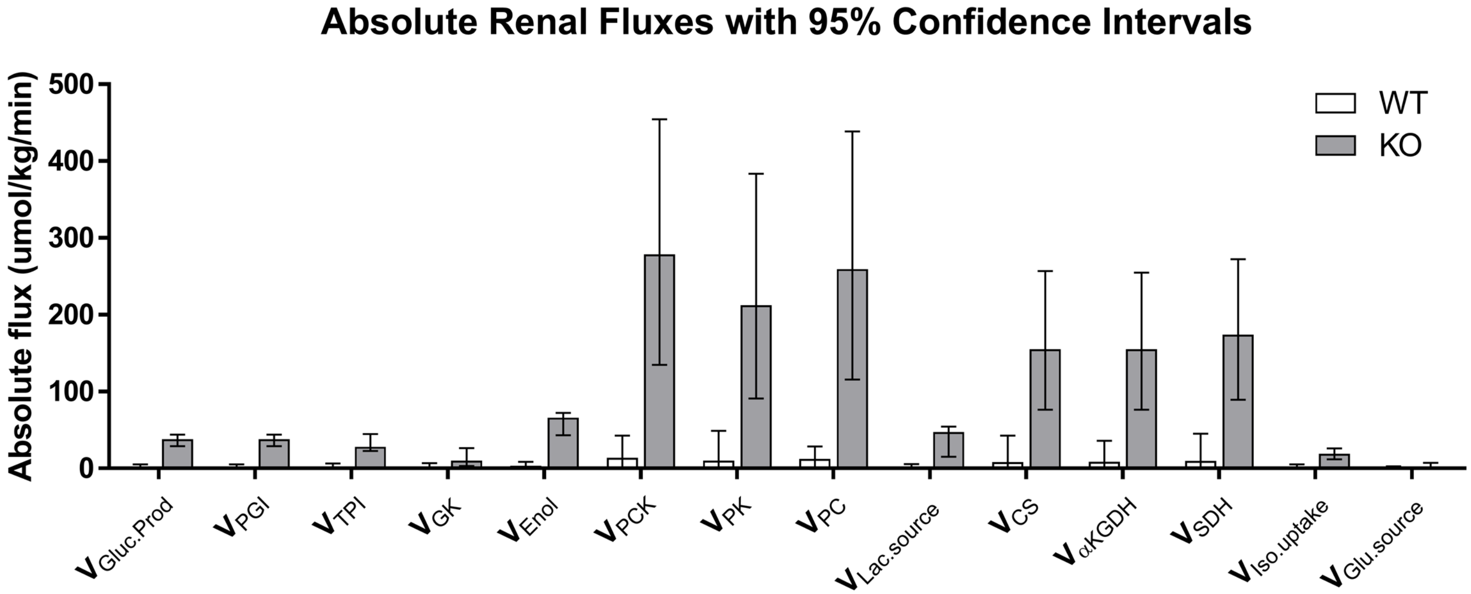

Figure S4. Mean hepatic and renal fluxes with 95% confidence intervals represented as error bars. Absolute **A.** hepatic and **B.** renal fluxes for WT (n=7) and KO (n=4) mice.

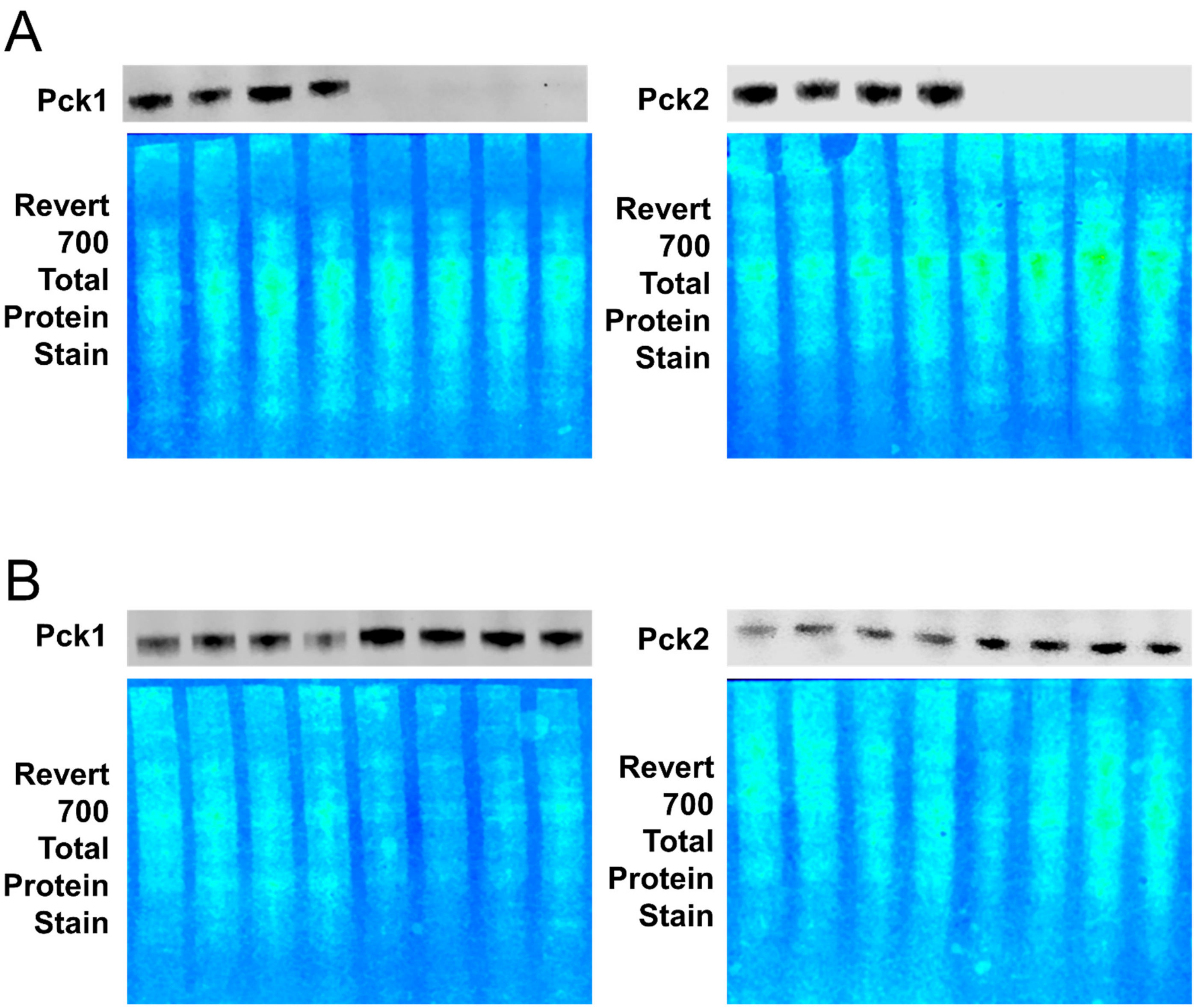

Figure S5. Protein expression of Pck1 (PEPCK-C) and Pck2 (PEPCK-M) in the liver and kidney. Total protein stain used to determine fold changes in protein expression of Pck1 and Pck2 in the **A.** liver and **B.** kidney of KO (n=4) and WT (n=4) mice.

Table S1. Dual organ metabolic reaction network for $^{2}H/^{13}C$ MFA.
Network maps of liver and kidney metabolism track carbon (uppercase) and hydrogen (lowercase) atoms through model reactions. Metabolites used to regress fluxes in both compartments are shown in Table S2. Metabolites in the liver, kidney, and plasma compartments are denoted as ".L", ".K", and ".P," respectively. Unenriched sources and sinks for "H" and "CO_2" are annotated as ".source" and ".sink", respectively. ^{2}H and ^{13}C isotopes are introduced into model reactions as "iso.uptake". The Dummy metabolite and reaction V_{Fixed} were used to constrain the relative glucose production rate to 100.

Hepatic Compartment	
**Flux**	**Reaction Network**
$V_{GlucProd.L}$	G6P.L (AaBbCcDdEeFfg) → Gluc.P (AaBbCcDdEeFfg) + Dummy
$V_{PYGL.L}$	Glycogen.L (AaBbCcDdEeFfg) + H (q) → G6P.L (AaBqCcDdEeFfg) + H (b)
$V_{PGI.L}$	F16BP.L (CcdBAaDeEfFgh) + H (q) → G6P.L (CcBqAaDeEfFgh) + H (d)
$V_{Ald.L}$	DHAP.L (AabBCcd) + GAP.L (DeEfFgh) → F16BP.L (CcdBAaDeEfFgh) + H (b)
$V_{TPI.L}$	DHAP.L (AaqBCcd) + H (z) ↔ GAP.L (AqBzCcd) + H (a)
$V_{G3PDH.L}$	G3P.L (AabBcCde) ↔ DHAP.L (AabBCde) + H (c)
$V_{GK.L}$	Glycerol.L (AabBcCde) → G3P.L (AabBcCde)
$V_{GAPDH.L}$	BPG.L (ABbCcd) + H (a) → GAP.L (AaBbCcd)
$V_{Enol.L}$	PEP.L (ABCcd) + H (b) → BPG.L (ABbCcd)
$V_{PCK.L}$	Oac.L (ABCabD) → PEP.L (ABCab) + CO2.L (D)
$V_{PK+ME.L}$	PEP.L (ABCab) + H (c) → Pyr.L (ABCabc)
$V_{PC.L}$	Pyr.L (ABCcde) + CO2.L (D) → Oac.L (ABCcdD) + H (e)
$V_{LDH.L}$	Lac.L (ABbCcde) ↔ Pyr.L (ABCcde) + H (b)
$V_{Lac\ Source.L}$	Lac.Source (ABbCcde) → Lac.L (ABbCcde)
$V_{CS.L}$	Oac.L (ABCcdD) + AcCoA.L (EFfgh) → Cit.L (DCcdBFfgEA) + H (h)
$V_{ICDH.L}$	Cit.L (ABabCDcdEF) + H (e) ↔ α-KG.L (ABCeaDcdE) + H (b) + CO2.L (F)
$V_{aKGDH.L}$	α-KG.L (ABCabDcdE) → SucCoA.L (BCabDcdE) + CO2.L (A)
$V_{SCS.L}$	SucCoA.L (ABabCcdD) → Suc.L (ABabCcdD)
$V_{SDH.L}$	Suc.L (ABabCcdD) ↔ Fum.L (ABaCdD) + H (b) + H (c)
$V_{FM.L}$	Fum.L (ABaCbD) + H (c) ↔ Mal.L (ABaCcbD)
$V_{MDH.L}$	Mal.L (ABaCbcD) ↔ Oac.L (ABCbcD) + H (a)
$V_{CO2\ Source.L}$	BicarbL.source (A) → CO2.L (A)
$V_{CO2\ Sink.L}$	CO2.L (A) → BicarbL.sink (A)
$V_{Iso.uptake.L}$	PropCoA.inf (ABabCcde) → PropCoA.L (ABabCcde)
$V_{PCC.L}$	PropCoA.L (ABabCcde) + CO2.L (D) → SucCoA.L (ACcdBabD) + H (e)
$V_{ALT.L}$	Ala.L (ABbCcde) + H (f) ↔ Pyr.L (ABCcdf) + H (b) + H (e)
$V_{GDH.L}$	Glu.L (ABeCabDcdE) ↔ α-KG.L (ABCabDcdE) + H (e)
$V_{Glu\ Source.L}$	Glu.Source (ABeCabDcdE) → Glu.L (ABeCabDcdE)

Renal Compartment	
**Flux**	**Reaction Network**
$V_{GlucProd.K}$	G6P.K (AaBbCcDdEeFfg) → Gluc.P (AaBbCcDdEeFfg) + Dummy
$V_{PGI.K}$	F16BP.K (CcdBAaDeEfFgh) + H (q) → G6P.K (CcBqAaDeEfFgh) + H (d)
$V_{Ald.K}$	DHAP.K (AabBCcd) + GAP.K (DeEfFgh) → F16BP.K (CcdBAaDeEfFgh) + H (b)
$V_{TPI.K}$	DHAP.K (AaqBCcd) + H (z) ↔ GAP.K (AqBzCcd) + H (a)
$V_{GAPDH.K}$	BPG.K (ABbCcd) + H (a) → GAP.K (AaBbCcd)
$V_{G3PDH.K}$	G3P.K (AabBcCde) ↔ DHAP.K (AabBCde) + H (c)
$V_{GK.K}$	Glycerol.K (AabBcCde) → G3P.K (AabBcCde)
$V_{Enol.K}$	PEP.K (ABCcd) + H (b) → BPG.K (ABbCcd)
$V_{PCK.K}$	Oac.K (ABCabD) → PEP.K (ABCab) + CO2.K (D)
$V_{PK+ME.K}$	PEP.K (ABCab) + H (c) → Pyr.K (ABCabc)
$V_{PC.K}$	Pyr.K (ABCcde) + CO2.K (D) → Oac.K (ABCcdD) + H (e)
$V_{LDH.K}$	Lac.K (ABbCcde) ↔ Pyr.K (ABCcde) + H (b)
$V_{Lac\ Source.K}$	Lac.Source (ABbCcde) → Lac.K (ABbCcde)
$V_{CS.K}$	Oac.K (ABCcdD) + AcCoA.K (EFfgh) → Cit.K (DCcdBFfgEA) + H (h)
$V_{ICDH.K}$	Cit.K (ABabCDcdEF) + H (e) ↔ α-KG.K (ABCeaDcdE) + H (b) + CO2.K (F)
$V_{aKGDH.K}$	α-KG.K (ABCabDcdE) → SucCoA.K (BCabDcdE) + CO2.K (A)
$V_{SCS.K}$	SucCoA.K (ABabCcdD) → Suc.K (ABabCcdD)
$V_{SDH.K}$	Suc.K (ABabCcdD) ↔ Fum.K (ABaCdD) + H (b) + H (c)
$V_{FM.K}$	Fum.K (ABaCbD) + H (c) ↔ Mal.K (ABaCcbD)
$V_{MDH.K}$	Mal.K (ABaCbcD) ↔ Oac.K (ABCbcD) + H (a)
$V_{CO2\ Source.K}$	BicarbK.source (A) → CO2.K (A)
$V_{CO2\ Sink.K}$	CO2.K (A) → BicarbL.sink (A)
$V_{Iso.uptake.K}$	PropCoA.inf (ABabCcde) → PropCoA.K (ABabCcde)
$V_{PCC.K}$	PropCoA.K (ABabCcde) + CO2.K (D) → SucCoA.K (ACcdBabD) + H (e)
$V_{ALT.K}$	Ala.K (ABbCcde) + H (f) ↔ Pyr.K (ABCcdf) + H (b) + H (e)
$V_{GDH.K}$	Glu.K (ABeCabDcdE) ↔ α-KG.K (ABCabDcdE) + H (e)
$V_{Glu\ Source.K}$	Glu.Source (ABeCabDcdE) → Glu.K (ABeCabDcdE)

**Transport Reactions**	
V_{66Gluc}	Gluc.inf (AaBbCcDdEeFfg) → Gluc.P (AaBbCcDdEeFfg)
V_{Hinf}	H.inf (a) → H (a)
V_{Hsink}	H → Sink
V_{Fixed}	Dummy → Sink

Table S2. GC-MS fragment ions of measured metabolites regressed using the metabolic model for MFA.
H_R and H_S refer to diastereotopic hydrogens attached to each carbon. Hydrogens marked with an asterisk (H*) are modeled as equivalent.

Metabolite	m/z	Derivative Formula	Carbons and Hydrogens												
Alanine	260	$C_{11}H_{26}O_2NSi_2$	C1	C2	H1	C3	H2*	H3*	H4*						
Alanine	232	$C_{10}H_{26}ONSi_2$		C2	H1	C3	H2*	H3*	H4*						
Glutamate	432	$C_{19}H_{42}O_4NSi_3$	C1	C2	H1	C3	$H2_R$	$H3_S$	C4	$H4_R$	$H5_S$	C5			
Glutamate	330	$C_{16}H_{36}O_2NSi_2$		C2	H1	C3	$H2_R$	$H3_S$	C4	$H4_R$	$H5_S$	C5			
Glycerol-3-Phosphate	571	$C_{20}H_{51}O_6Si_4P$	C1	$H1_R$	$H2_S$	C2	H3	C3	$H4_R$	$H5_S$					
Glucose	370	$C_{17}H_{24}O_8N$	C1		C2	H2	C3	H3	C4	H4	C5	H5			
Glucose	301	$C_{14}H_{21}O_7$	C1	H1	C2	H2	C3	H3	C4	H4	C5	H5	C6	$H6_R$	$H7_S$
Glucose	284	$C_{13}H_{18}O_6N$	C1		C2	H2	C3	H3	C4	H4					
Glucose	259	$C_{12}H_{19}O_6$							C4	H4	C5	H5	C6	$H6_R$	$H7_S$
Glucose	173	$C_8H_{13}O_4$									C5	H5	C6	$H6_R$	$H7_S$
Glucose	145	$C_6H_{11}O_3N$	C1	H1	C2	H2									
Lactate	261	$C_{11}H_{25}O_3Si_2$	C1	C2	H1	C3	H2*	H3*	H4*						
Lactate	233	$C_{10}H_{25}O_2Si_2$		C2	H1	C3	H2*	H3*	H4*						
Urea	231	$C_{13}H_{32}N_2OSi_2$	C1												

Table S3. Metabolic fluxes in the liver and kidneys of WT and KO mice (Related to Fig. 3)
Absolute fluxes (mean ± SEM) in the hepatic and renal compartment of WT (n=7) and KO (n=4) mice. Exchange fluxes have been normalized to a scale of 0−100 by dividing each exchange flux (V_{exch}) by the sum V_{exch}+$V_{Gluc.Prod}$, where $V_{Gluc.Prod}$ is the combined glucose production flux from liver and kidneys. Significant flux differences between WT and KO mice are marked with an asterisk, analyzed by a 2-tailed *t* test where * *P* < 0.05.

	WT	KO
**Liver Compartment**		
$V_{GlucProd.L}$*	69 ± 5	21 ± 5
$V_{PYGL.L}$	8 ± 1	10 ± 4
$V_{PGI.L}$*	61 ± 5	11 ± 4
$V_{Ald.L}$*	61 ± 5	11 ± 4
$V_{TPI.L}$*	47 ± 3	4 ± 3
$V_{TPI.L}$ exchange	78 ± 7	100 ± 1
$V_{G3PDH.L}$*	15 ± 4	14 ± 2
$V_{G3PDH.L}$ exchange	12 ± 3	32 ± 23
$V_{GK.L}$	15 ± 4	14 ± 2
$V_{GAPDH.L}$*	107 ± 7	8 ± 6
$V_{Enol.L}$*	107 ± 7	8 ± 6
$V_{PCK.L}$*	347 ± 40	12 ± 11
$V_{PK+ME.L}$*	240 ± 37	5 ± 5
$V_{PC.L}$*	307 ± 37	10 ± 9
$V_{LDH.L}$*	45 ± 9	1 ± 1
$V_{LDH.L}$ exchange*	54 ± 10	3 ± 2
$V_{Lac\ Source.L}$*	45 ± 9	1 ± 1
$V_{CS.L}$*	83 ± 8	17 ± 12
$V_{ICDH.L}$*	83 ± 8	17 ± 12
$V_{ICDH.L}$ exchange*	100 ± 1	26 ± 25
$V_{aKGDH.L}$*	104 ± 10	17 ± 12
$V_{SCS.L}$*	124 ± 11	20 ± 14
$V_{SDH.L}$*	124 ± 11	20 ± 14
$V_{SDH.L}$ exchange	12 ± 9	27 ± 25
$V_{FM.L}$*	124 ± 11	20 ± 14
$V_{Fum.L}$ exchange	83 ± 11	44 ± 22
$V_{MDH.L}$*	124 ± 11	20 ± 14
$V_{MDH.L}$ exchange*	87 ± 5	25 ± 25

	WT	KO
$V_{CO2\ Source.L}$*	591 ± 87	132 ± 62
$V_{CO2\ Sink.L}$*	798 ± 93	166 ± 85
$V_{Iso.uptake.L}$*	20 ± 2	3 ± 2
$V_{PCC.L}$*	20 ± 2	3 ± 2
$V_{ALT.L}$*	23 ± 10	5 ± 5
$V_{ALT.L}$ exchange	46 ± 13	76 ± 25
$V_{GDH.L}$*	23 ± 10	1 ± 1
$V_{GDH.L}$ exchange*	100 ± 1	25 ± 25
$V_{Glu\ Source.L}$*	21 ± 5	1 ± 1
**Renal Compartment**		
$V_{GlucProd.K}$*	2 ± 2	39 ± 7
$V_{PGI.K}$*	2 ± 2	39 ± 7
$V_{Ald.K}$*	2 ± 2	39 ± 7
$V_{TPI.K}$*	2 ± 2	29 ± 5
$V_{TPI.K}$ exchange	58 ± 16	33 ± 10
$V_{GAPDH.K}$*	4 ± 4	67 ± 12
$V_{G3PDH.K}$*	1 ± 1	11 ± 3
$V_{G3PDH.K}$ exchange	1 ± 1	2 ± 1
$V_{GK.K}$*	1 ± 1	11 ± 3
$V_{Enol.K}$*	4 ± 4	67 ± 12
$V_{PCK.K}$*	14 ± 14	279 ± 34
$V_{PK+ME.K}$*	11 ± 11	213 ± 27
$V_{PC.K}$*	13 ± 13	260 ± 34
$V_{LDH.K}$*	3 ± 3	48 ± 11
$V_{LDH.K}$ exchange*	3 ± 3	50 ± 6
$V_{Lac\ Source.K}$*	3 ± 3	48 ± 11
$V_{CS.K}$*	9 ± 9	156 ± 18
$V_{ICDH.K}$*	9 ± 9	156 ± 18
$V_{ICDH.K}$ exchange	100 ± 1	50 ± 27
$V_{aKGDH.K}$*	9 ± 9	156 ± 18
$V_{SCS.K}$*	10 ± 10	175 ± 18
$V_{SDH.K}$*	10 ± 10	175 ± 18

	WT	KO
$V_{SDH.K}$ exchange	1 ± 1	1 ± 1
$V_{FM.K}$*	10 ± 10	175 ± 18
$V_{Fum.K}$ exchange	93 ± 7	100 ± 1
$V_{MDH.K}$*	10 ± 10	175 ± 18
$V_{MDH.K}$ exchange*	12 ± 12	96 ± 3
$V_{CO2\ Source.K}$*	87 ± 87	1019 ± 284
$V_{CO2\ Sink.K}$*	104 ± 104	1329 ± 309
$V_{Iso.uptake.K}$*	2 ± 2	20 ± 2
$V_{PCC.K}$*	2 ± 2	20 ± 2
$V_{ALT.K}$	1 ± 1	1 ± 1
$V_{ALT.K}$ exchange	1 ± 1	1 ± 1
$V_{GDH.K}$	1 ± 1	1 ± 1
$V_{GDH.K}$ exchange*	16 ± 11	76 ± 25
$V_{Glu\ Source.K}$	1 ± 1	1 ± 1
